# Supplementary material for: Determination of ubiquitin fitness landscapes under different chemical stresses in a classroom setting
Source: eLife. 2016 Apr 25;5:e15802. doi: 10.7554/eLife.15802 (PMC4862753; doi:10.7554/eLife.15802)
Supplement: Figure 9—source data 1. — Mutants in the shared response were determined by fitting a line to the fitness scores. The distance from each point to that line was calculated. If the distance was less than 0.1 and the average Δ (DMSO - Perturbation) fitness was less than -0.2 the mutant was considered part of the shared response. E1 activity relative to WT Ub (Roscoe and Bolon, 2014) is listed and may explain the sensitization of some of the shared response mutants. DOI: http://dx.doi.org/10.7554/eLife.15802.017 [file elife-15802-fig9-data1.docx]

| **Wild Type** | **Mutant** | **Type** | **Average Δ (DMSO - Perturbation) fitness** | **Relative E1 reactivity** |
| --- | --- | --- | --- | --- |
| Gln2 | Aspartate | Polar to negative | -0.46 | 1.06 |
| Val5 | Aspartate | Hydrophobic to negative | -0.20 | 0.00 |
| Lys6 | Proline | Positive to proline | -0.35 | 0.08 |
| Thr7 | Methionine | Polar to hydrophobic | -0.41 | 1.01 |
| Thr7 | Glutamine | Polar to negative | -0.25 | 1.02 |
| Leu8 | Tyrosine | Hydrophobic to aromatic | -0.25 | 0.84 |
| Leu8 | Histidine | Hydrophobic to positive | -0.28 | 0.66 |
| Leu8 | Aspartate | Hydrophobic to negative | -0.72 | 0.21 |
| Thr12 | Valine | Polar to hydrophobic | -0.39 | 0.95 |
| Ile13 | Tyrosine | Hydrophobic to aromatic | -0.31 | 0.95 |
| Val26 | Arginine | Hydrophobic to positive | -0.31 | 0.09 |
| Lys27 | Serine | Positive to polar | -0.30 | 0.55 |
| Ile30 | Glycine | Hydrophobic to glycine | -0.28 | 0.46 |
| Asp32 | Phenylalanine | Negative to aromatic | -0.26 | 1.00 |
| Asp32 | Isoleucine | Negative to hydrophobic | -0.29 | 0.99 |
| Glu34 | Leucine | Glycine to hydrophobic | -0.32 | 1.03 |
| Pro37 | Tyrosine | Proline to aromatic | -0.31 | 0.96 |
| Gln41 | Proline | Polar to proline | -0.24 | 0.42 |
| Arg42 | Cystine | Positive to cystine | -0.27 | -0.13 |
| Arg42 | Proline | Positive to proline | -0.23 | 0.31 |
| Leu43 | Tyrosine | Hydrophobic to aromatic | -0.32 | 0.87 |
| Gly47 | Phenylalanine | Glycine to aromatic | -0.38 | 0.14 |
| Gly47 | Threonine | Glycine to polar | -0.21 | 0.47 |
| Gln49 | Tyrosine | Polar to aromatic | -0.28 | -0.61 |
| Leu50 | Glycine | Hydrophobic to glycine | -0.40 | 0.15 |
| Asp58 | Tyrosine | Negative to aromatic | -0.26 | 0.94 |
| Asp58 | Proline | Negative to proline | -0.30 | 0.52 |
| Ile61 | Tyrosine | Hydrophobic to aromatic | -0.36 | 0.20 |
| Ile61 | Glycine | Hydrophobic to glycine | -0.26 | 0.06 |
| Ile61 | Lysine | Hydrophobic to positive | -0.28 | -0.02 |
| Thr66 | Tyrosine | Polar to aromatic | -0.37 | 0.93 |
| Thr66 | Isoleucine | Polar to hydrophobic | -0.24 | 0.94 |
| Thr66 | Arginine | Polar to positive | -0.24 | 0.98 |
| Leu67 | Glycine | Hydrophobic to glycine | -0.21 | 0.90 |
| Leu69 | Arginine | Hydrophobic to positive | -0.27 | 0.94 |
| Val70 | Tyrosine | Hydrophobic to aromatic | -0.25 | -0.07 |
| Leu71 | Serine | Hydrophobic to polar | -0.22 | 1.02 |
| Arg74 | Isoleucine | Positive to hydrophobic | -0.37 | 1.00 |
| Gly75 | Phenylalanine | Glycine to aromatic | -0.41 | 0.11 |
| Gly75 | Valine | Glycine to hydrophobic | -0.26 | 0.11 |
| Gly75 | Asparagine | Glycine to polar | -0.24 | 0.17 |
