# Supplementary material for: Determination of ubiquitin fitness landscapes under different chemical stresses in a classroom setting
Source: eLife. 2016 Apr 25;5:e15802. doi: 10.7554/eLife.15802 (PMC4862753; doi:10.7554/eLife.15802)
Supplement: Figure 9—source data 2. — Perturbation specific mutations were determined by fitting a line to the delta (DMSO - perturbation) fitness scores. The distance from each point to that line was calculated. If the distance was greater than 0.35 the mutant was classified as perturbation specific. Mutants with high experimental errors were deemed outliers and removed from this list. DOI: http://dx.doi.org/10.7554/eLife.15802.018 [file elife-15802-fig9-data2.docx]

| **Wild Type** | **Mutant** | **Type** | **Notes** | **Δ fitness score in Caffeine** | **SD** | **Δ fitness score in DTT** | **SD** | **Δ fitness score in HU** | **SD** |
| --- | --- | --- | --- | --- | --- | --- | --- | --- | --- |
| Lys11 | Asparagine | Positive to polar | Lys11 linked poly-Ub | 0.55 | 0.04 | -0.32 | 0.03 | -0.03 | 0.03 |
| Thr14 | Valine | Polar to hydrophobic | Surface exposed beta strand, adjacent to Phe4 | 0.16 | 0.0 | -0.01 | 0.17 | -0.33 | 0.03 |
| Ser20 | Tyrosine | Polar to aromatic | Surface exposed loop | 0.5 | 0.07 | -0.33 | 0.02 | -0.01 | 0.09 |
| Asp21 | Phenylalanine | Negative to aromatic | Surface exposed loop | 0.11 | 0.35 | -0.41 | 0.16 | -0.01 | 0.19 |
| Thr22 | Histidine | Polar to positive | Putative helix cap | 0.51 | No Estimate | -0.45 | No Estimate | -0.15 | No Estimate |
| Gln40 | Asparagine | Shortened by one carbon | Surface exposed loop | 0.20 | 0.27 | -0.49 | 0.17 | -0.09 | 0.06 |
| Leu56 | Methionine | Extension of hydrophobic group | Core residue | 0.57 | 0.03 | -0.48 | 0.0 | -0.19 | 0.0 |
| His68 | Tyrosine | Positive to aromatic | Surface exposed beta strand | 0.17 | 0.09 | -0.30 | 0.17 | -0.28 | 0.11 |
