# Supplementary material for: Determination of ubiquitin fitness landscapes under different chemical stresses in a classroom setting
Source: eLife. 2016 Apr 25;5:e15802. doi: 10.7554/eLife.15802 (PMC4862753; doi:10.7554/eLife.15802)
Supplement: Figure 9—source data 3. — DOI: http://dx.doi.org/10.7554/eLife.15802.019 [file elife-15802-fig9-data3.docx]

| **Mutant** | **Perturbation** | **Average of the barcode**  **fitness scores** | **Standard**  **deviation of barcode scores** | **Number of barcodes** | **Initial number of observations of each barcode** |
| --- | --- | --- | --- | --- | --- |
| Lys11Asn | Caffeine | 0.60 | 0.04 | 4 | 140, 24, 93, 105 |
|  | DTT | -0.27 | 0.03 | 4 | 1120, 188, 647, 698 |
|  | HU | 0.02 | 0.03 | 5 | 485, 1878, 69, 299, 287 |
| Glu64Arg | Caffeine | < -0.50 | 0.105 | 25 | 115, 3, 32, 91, 12, 174, 37, 27, 7, 4, 101, 15, 18, 66, 8, 52, 21, 34, 36, 4, 40, 14, 20, 36, 24 |
|  | DTT | -0.24 | 0.160 | 28 | 10, 102, 28, 38, 29, 101, 7, 172, 18, 39, 113, 21, 18, 65, 15, 9, 3, 68, 30, 44, 41, 8, 34, 37, 11, 27, 18, 24 |
|  | HU | -0.48 | 0.155 | 29 | 6, 96, 24, 28, 15, 83, 8, 153, 46, 3, 20, 7, 3, 76, 16, 12, 42, 5, 13, 51, 30, 31, 15, 15, 25, 9, 17, 11, 16 |
| His68Tyr | Caffeine | -0.03 | 0.09 | 3 | 16, 24, 18 |
|  | DTT | < -0.50 | 0.17 | 3 | 23, 26, 26 |
|  | HU | -0.48 | 0.111 | 3 | 15, 12, 20 |
